# Plant cell wall in pathogenesis, parasitism and symbiosis

**DOI:** 10.3389/fpls.2014.00612

**Published:** 2014-11-06

**Authors:** Vincenzo Lionetti, Jean-Pierre Métraux

**Affiliations:** ^1^Dipartimento di Biologia e Biotecnologie “Charles Darwin,” Sapienza Università di RomaRome, Italy; ^2^Department of Biology, University of FribourgFribourg, Switzerland

**Keywords:** plant cell wall integrity, susceptibility factors, methanol, callose, plant symbioses, cell wall degrading enzymes, plant pathogens, plant parasitic nematode

The cell wall is the foremost interface at which interactions take place between plants and a wide range of other organisms including insects, nematodes, pathogenic, or symbiotic microorganisms.

This Research Topic includes Mini Reviews, Reviews, Original Research Articles, and Perspective Articles that provide novel insights and detailed overviews on the dynamics of the plant cell wall in plant defense, parasitism, and symbiosis and describes experimental approaches to study plant cell wall modifications occurring during interaction of plants with different organisms.

The cuticle represents the first cell wall layer encountered by pathogens. Serrano and collaborators describe the influence of the cuticle components on microbial development during pathogenesis (Serrano et al., 2014). The authors highlight also the alterations of the cuticle that induce defense responses against necrotrophs. Once the microbe has penetrated, the cell wall becomes a battleground where plants and pathogens attempt to prevail. Necrotrophic fungi produce several cell wall degrading enzymes (CWDEs) to degrade plant cell wall polysaccharides that favor plant colonization. Blanco-Ulate and co-authors present a genome-wide transcriptional profiling of *Botrytis cinerea* CWDEs expressed during infection of important nutritional resources such as lettuce leaves, ripe tomato fruit, and grape berries (Blanco-Ulate et al., 2014).

Plants develop different cell wall related systems for sensing intruders. Malinovsky and co-workers provide an overview of the cell surface pattern recognition receptors (PRRs) that perceive a diverse set of microbial molecules referred to as microbial/pathogen-associated-molecular patterns (MAMPs/PAMPs) and trigger immune responses (Malinovsky et al., 2014). Bellincampi and co-authors illustrate the mechanisms of sensing the alteration of cell wall integrity (CWI) during biotic stress and explain how cell walls can be a source of the so-called damage-associated molecular patterns (DAMPs) (Bellincampi et al., 2014). Three manuscripts of this research topic deal with specific mechanisms of perception and signaling of cell wall damage and modulation of plant immunity. Emerging evidence indicates that the Arabidopsis plasma membrane-localized protein NDR1 (NON-RACE-SPECIFIC DISEASE RESISTANCE 1) functions as key signaling component of the loss of membrane-cell wall adhesions during pathogen infection. The research article by Lu and co-workers reports on the identification of a citrus ortholog CsNDR1 that, when overexpressed in Arabidopsis, improves disease resistance to *Pseudomonas syringae* and *Hyaloperonospora arabidopsidis* (Lu et al., 2013). Komarova and co-authors discuss on the role of methanol as signal in plant immunity (Komarova et al., 2014). Methanol (MeOH) can be released by plant pectin methylesterases, induced by the mechanical damage of plant tissues during microbe penetration. The emission of MeOH by a wounded plant can have a priming effect, enhancing the resistance of the non-wounded, neighboring receiver plants to pathogens. Tauzin and Giardina review the emerging argument of the “sweet immunity” (Tauzin and Giardina, 2014). The authors describe the involvement of sucrose and cell wall invertases as priming agents important for triggering an appropriate defense responses during pathogen invasion.

During pathogen infection plants can repair to the loss of CWI by activating cell wall reinforcing mechanisms (Bellincampi et al., 2014). At sites of interaction with intruding microbial pathogens the cell wall is actively reinforced through the deposition of cell wall appositions, so-called papillae. Voigt present a perspective article that discusses the possible roles of the (1,3)-β-glucan callose and the other papillae components in plant defense (Voigt, 2014). Lignin is both pathogen-induced and developmentally deposited in the secondary thickened cell wall. In their review article Miedes and collaborators update us on the effect of altered lignin amount and composition on pathogen infection and spread (Miedes et al., 2014).

Pathogens, parasites, and symbionts may exploit the host cell wall metabolism to support the colonization of their hosts (Bellincampi et al., 2014). An important remodeling of the plant cell wall is also exploited during plant parasitism by cyst nematodes. Bohlmann and Sobczak present a detailed overview of the cell wall degrading and modifying enzymes that nematodes produce during migration through the root, and the cell wall modifications occurring during syncytium development due to the cell wall synthetizing, modifying, and degrading proteins of the plant (Bohlmann and Sobczak, 2014). Two articles of this research topic report on the cell wall remodeling during mutualistic symbiosis of plants with different microorganisms, useful to establish an intimate interface for developmental coordination and nutrient exchange. Rich and co-workers put the emphasis on modifications of the cell wall compartment during penetration and establishment of the symbiotic interface in root nodule symbiosis and arbuscular mycorrhizae (Rich et al., 2014). Genetic, genomic, and transcriptomic analysis related to symbiotic signaling, cell wall loosening and penetration, and the nutrition of the microbial endosymbionts are assessed. The current knowledge on the dynamics of plant and fungal walls in ectomycorrhizae and arbuscular-mycorrhizae is presented in the review article provided by Balestrini and Bonfante (2014). The cumulative evidence suggests that the plant CWDEs play a central role in the softening and remodeling of the cell wall during symbioses.

The analysis of the complex and dynamic modification of cell walls in response to pathogens is a technical challenge. Xia and co-authors summarize experimental approaches based on cell imaging, spectroscopic analyses and metabolic profiling techniques (Xia et al., 2014). The review by Delaunois and co-worker provides an insight into the modulation of the apoplastic protein patterns during pathogen infection (Delaunois et al., 2014). Super-resolution microscopy using microscopy combined with specific and efficient labeling techniques yield information on three-dimensional modifications of cell wall polymers (i.e., callose-cellulose network) at the site of attempted microbial penetration (Voigt, 2014). Carbohydrate microarrays, combining the specificity of monoclonal antibodies and carbohydrate binding modules with the multiplexed analysis capacity of microarrays, represent a promising technique to study the changes in cell wall micro-domains during plant/biotic interaction (Malinovsky et al., 2014).

These outstanding contributions reflect the considerable progress that has been made in the understanding the relationships at the cell wall interface between plants and other organisms. These publications represent our current knowledge on important physiological processes and provide food for thought for future research. As a concluding remark, we are grateful to the authors who responded enthusiastically to the call and reviewers for their valuable feedback to ensure the highest quality in the articles.

## Conflict of interest statement

The authors declare that the research was conducted in the absence of any commercial or financial relationships that could be construed as a potential conflict of interest.

## References

[B1] Balestrini R. Bonfante P. (2014). Cell wall remodeling in mycorrhizal symbiosis: a way towards biotrophism. Front. Plant Sci. 5:237. 10.3389/fpls.2014.00237 24926297 PMC4044974

[B2] Bellincampi D. Cervone F. Lionetti V. (2014). Plant cell wall dynamics and wall-related susceptibility in plant-pathogen interactions. Front. Plant Sci. 5:228. 10.3389/fpls.2014.00228 24904623 PMC4036129

[B3] Blanco-Ulate B. Morales-Cruz A. Amrine K. C. H. Labavitch J. M. Powell A. L. T. Cantu D. (2014). Genome-wide transcriptional profiling of *Botrytis cinerea* genes targeting plant cell walls during infections of different hosts. Front. Plant Sci. 5:435. 10.3389/fpls.2014.00435 25232357 PMC4153048

[B4] Bohlmann H. Sobczak M. (2014). The plant cell wall in the feeding sites of cyst nematodes. Front. Plant Sci. 5:89. 10.3389/fpls.2014.00089 24678316 PMC3958752

[B5] Delaunois B. Jeandet P. Clement C. Baillieul F. Dorey S. Cordelier S. (2014). Uncovering plant-pathogen crosstalk through apoplastic proteomic studies. Front. Plant Sci. 5:249. 10.3389/fpls.2014.00249 24917874 PMC4042593

[B6] Komarova T. V. Sheshukova E. V. Dorokhov Y. L. (2014). Cell wall methanol as a signal in plant immunity. Front. Plant Sci. 5:101. 10.3389/fpls.2014.00101 24672536 PMC3957485

[B7] Lu H. Zhang C. Albrecht U. Shimizu R. Wang G. F. Bowman K. D. (2013). Overexpression of a citrus NDR1 ortholog increases disease resistance in Arabidopsis. Front. Plant Sci. 4:157. 10.3389/fpls.2013.00157 23761797 PMC3669760

[B8] Malinovsky F. G. Fangel J. U. Willats W. G. T. (2014). The role of the cell wall in plant immunity. Front. Plant Sci. 5:178. 10.3389/fpls.2014.00178 24834069 PMC4018530

[B9] Miedes E. Vanholme R. Boerjan W. Molina A. (2014). The role of the secondary cell wall in plant resistance to pathogens. Front. Plant Sci. 5:358. 10.3389/fpls.2014.00358 25161657 PMC4122179

[B10] Rich M. K. Schorderet M. Reinhardt D. (2014). The role of the cell wall compartment in mutualistic symbioses of plants. Front. Plant Sci. 5:238. 10.3389/fpls.2014.00238 24917869 PMC4041022

[B11] Serrano M. Coluccia F. Torres M. L'Haridon F. Metraux J. P. (2014). The cuticle and plant defense to pathogens. Front. Plant Sci. 5:274. 10.3389/fpls.2014.00274 24982666 PMC4056637

[B12] Tauzin A. S. Giardina T. (2014). Sucrose and invertases, a part of the plant defense response to the biotic stresses. Front. Plant Sci. 5:293. 10.3389/fpls.2014.00293 25002866 PMC4066202

[B13] Voigt C. A. (2014). Callose-mediated resistance to pathogenic intruders in plant defense-related papillae. Front. Plant Sci. 5:168. 10.3389/fpls.2014.00168 24808903 PMC4009422

[B14] Xia Y. Petti C. Williams M. A. DeBolt S. (2014). Experimental approaches to study plant cell walls during plant-microbe interactions. Front. Plant Sci. 5:540. 10.3389/fpls.2014.00540 25352855 PMC4196508

